# COVID‐19 infection, progression, and vaccination: Focus on obesity and related metabolic disturbances

**DOI:** 10.1111/obr.13313

**Published:** 2021-07-16

**Authors:** Annemarie J. F. Westheim, Albert V. Bitorina, Jan Theys, Ronit Shiri‐Sverdlov

**Affiliations:** ^1^ Department of Precision Medicine, GROW‐Research School for Oncology and Reproduction Maastricht University Medical Center+ Maastricht The Netherlands; ^2^ Department of Molecular Genetics, NUTRIM‐School of Nutrition and Translational Research in Metabolism Maastricht University Medical Center+ Maastricht The Netherlands

**Keywords:** COVID‐19, metabolic syndrome, obesity, severe acute respiratory syndrome coronavirus 2

## Abstract

Coronaviruses are constantly circulating in humans, causing common colds and mild respiratory infections. In contrast, infection with the novel severe acute respiratory syndrome coronavirus 2 (SARS‐CoV‐2), responsible for coronavirus disease‐2019 (COVID‐19), can cause additional severe complications, particularly in patients with obesity and associated metabolic disturbances. Obesity is a principal causative factor in the development of the metabolic syndrome; a series of physiological, biochemical, clinical, and metabolic factors that increase the risk of obesity‐associated diseases. “Metabolically unhealthy” obesity is, in addition to metabolic disturbances, also associated with immunological disturbances. As such, patients with obesity are more prone to develop serious complications from infections, including those from SARS‐CoV‐2. In this review, we first describe how obesity and related metabolic disturbances increase the risk of SARS‐CoV‐2 infection. Then, mechanisms contributing to COVID‐19 complications and poor prognosis in these patients are discussed. Finally, we discuss how obesity potentially reduces long‐term COVID‐19 vaccination efficacy. Despite encouraging COVID‐19 vaccination results in patients with obesity and related metabolic disturbances in the short‐term, it is becoming increasingly evident that long‐term COVID‐19 vaccination efficacy should be closely monitored in this vulnerable group.

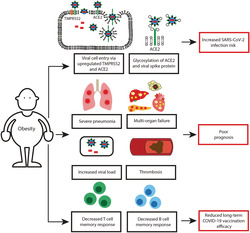

**Abbreviations**
ACE2: angiotensin‐converting enzyme 2
AID: activation‐induced cytidine deaminase
ALP: alkaline phosphatase
ALT: alanine aminotransferase
AMPK: AMP‐activated protein kinase
Ang II: angiotensin II
Ang (1‐7): angiotensin‐(1‐7)
ASC: apoptosis‐associated speck‐like protein containing a CARD
AST: aspartate aminotransferase
AT1R: angiotensin 1 receptor
AT2R: angiotensin 2 receptor
B_m_ cells: memory B cells
COPD: chronic obstructive pulmonary disease
COVID‐19: coronavirus disease‐2019
DIO: diet induced obesity
ECs: endothelium cells
EMA: European Medicine Agency
FDA: US Food and Drug Administration
HAI: hemagglutination inhibition
HDL: high‐density lipoproteins
HIF‐1α: hypoxia‐inducible factor‐1α
ICU: intensive care unit
IFN: interferons
Ig: immunoglobulin
IL‐6: interleukin 6
MCP1: monocyte chemoattractant protein 1
MERS‐CoV: Middle East respiratory syndrome coronavirus
MetS: metabolic syndrome
MGRA: MAS1 proto‐oncogene G protein‐coupled receptor
mTORC: mammalian target of rapamycin complex 1
mtROS: mitochondrial reactive oxygen species
NAFLD: non‐alcohol fatty liver disease
NASH: non‐alcoholic steatohepatitis
NF‐kB: nuclear factor kappa‐light‐chain‐enhancer of activated B cells
NLRP3: NLR family pyrin domain containing 3
oxLDL: oxidized low‐density lipoproteins
PAI‐1: plasminogen activator inhibitor‐1
PD‐1: programmed cell death protein 1
PD‐L1: programmed death‐ligand 1
PERK: PKR‐like endoplasmic reticulum kinase
PKR: protein kinase R
R: reproductive
RAAS: renin‐angiotensin aldosterone system
SARS‐CoV: syndrome coronavirus
SARS‐CoV‐2: severe acute respiratory syndrome coronavirus 2
T_em_ cells: effector memory T cells
TMPRSS2: Transmembrane Protease Serine 2
TNFα: tumor necrosis factor α
VOC: Variant of Concern 202012/01

## 1 INTRODUCTION

Coronavirus disease‐2019 (COVID‐19) caused by the novel severe acute respiratory syndrome coronavirus 2 (SARS‐CoV‐2) has taken the world by storm. Detected in more than 200 countries, with the number of confirmed cases already exceeding 114 million and deaths tallying at 2,5 million worldwide, it is one of the worst disasters of modern times.^1^

Viruses are grouped based on the type of genetic material they carry. DNA viruses have integrated large host DNA sequences in their genome throughout their evolution, which consequently allows for exploitation of host cell metabolism in order to promote viral replication. In contrast, RNA viruses such as respiratory syncytial virus, (para)influenza virus, metapneumovirus, rhinovirus, and coronavirus have smaller genomes consisting of RNA sequences encoding only a few proteins which are unfamiliar to the host. Despite this, RNA viruses can still manipulate host cells through other mechanisms to induce viral replication.^2^ Coronaviruses are responsible for 10%–30% of the common colds in humans.^3^ For example, the HCoV‐229E and HCoV‐OC43 viruses, regularly flaring up in temperate climate countries during winter or early spring, cause respiratory infections with mild clinical symptoms such as nasal obstruction, rhinorrhea, sneezing, sore throat, and cough.^4^, ^5^ In contrast, other coronaviruses such as severe acute respiratory syndrome coronavirus (SARS‐CoV) and Middle East respiratory syndrome coronavirus (MERS‐CoV) frequently cause serious respiratory illness.^6^, ^7^ The clinical course of SARS and MERS is remarkably similar, although subtle differences do exist such as the incidence of acute respiratory distress syndrome and the percentage of patients with comorbidities being higher in MERS then in SARS. In line, the mortality rate of MERS (36%) is higher compared with SARS (10%).^8^ Both, SARS‐CoV and MERS‐CoV are zoonotic coronaviruses that cross from animals to humans. Similarly, the novel highly pathogenic coronavirus, named SARS‐CoV‐2, is also zoonotic.^9^, ^10^ Sequencing data show a high resemblance between SARS‐CoV and SARS‐CoV‐2.^11^ Yet the higher reproductive (R) number of SARS‐CoV‐2 (2.87–3.44)^12^ compared with SARS‐CoV (1.7–1.9)^13^ indicates the higher pandemic potential of SARS‐CoV‐2, as also reflected in the rapid global spread of SARS‐CoV‐2 numbering in millions, whereas the absolute number of SARS‐CoV cases was only 8096.^1^, ^12^, ^13^, ^14^ Alarmingly, more contagions mutated SARS‐CoV‐2 variants have been detected in several countries. For example, the novel SARS‐CoV‐2 lineage designated as Variant of Concern 202012/01 (VOC), originally detected in England, has a substantial transmission advantage compared with the non‐VOC lineage with an estimated difference in R‐number ranging between 0.4 and 0.7 in a period of high levels of social distancing.^15^ In the earliest stages of the current COVID‐19 pandemic, it was thought that mostly the elderly, aged >60, were at risk of poor prognosis upon SARS‐CoV‐2 infection. However, it has become apparent that also patients aged <60 years are at risk of developing severe illness, particularly when they suffer from obesity.^16^

Notably, 10%–25% of the individuals with obesity are not affected by metabolic disturbances,^17^, ^18^, ^19^ whereas in the other individuals the abnormal or excessive fat accumulation presents a risk to health. Obesity is a principal causative factor in the development of the metabolic syndrome (MetS). MetS is defined as a series of physiological, biochemical, clinical, and metabolic factors that increase the risk of obesity‐associated diseases. These risk factors include central obesity with a focus on waist circumference, insulin resistance, hypertension, increased plasma triglycerides and reduced plasma high‐density lipoproteins (HDL).^20^ Though other abnormalities have also been associated with MetS, the presence of three of the five previously mentioned components is considered sufficient for a diagnosis of this syndrome, especially when associated with visceral obesity.^21^ Obesity, accompanied by the characteristics of MetS, constitutes the greatest threat to global health, affecting 20%–25% of the adult population.^21^ These patients are at increased risk of developing cardiovascular diseases, type 2 diabetes, and non‐alcoholic steatohepatitis (NASH).^22^ “Metabolically unhealthy” obesity is, in addition to metabolic disturbances, also associated with immunological disturbances, such as increased systemic leukocyte numbers and increased pro‐inflammatory plasma cytokine levels. This chronic low‐grade inflammatory state disrupts the immune response in patients with obesity. In that context, substantial evidence indicates a link between obesity and reduced host defense. For example, obesity increases the susceptibility for postoperative and nosocomial infections, and individuals with obesity are more prone to develop serious complications from common infections.^23^, ^24^ Moreover, during influenza pandemics (e.g., H1N1), individuals with obesity were overrepresented at the intensive care unit (ICU) and needed longer duration of mechanical ventilation compared with individuals with a healthy weight.^25^, ^26^ Diabetes, a frequently occurring obesity‐related metabolic comorbidity, actually tripled the risk of hospitalization after H1N1 infection and even quadrupled the risk of ICU admission once hospitalized.^27^ Additionally, obesity is associated with a reduced immunogenicity in response to vaccination for hepatitis B, tetanus, and influenza.^28^, ^29^ In line with these data, it is not surprising that patients suffering from obesity and related metabolic disturbances have poorer prognoses upon infection with the highly pathogenic coronaviruses SARS‐CoV,^30^ MERS‐CoV,^30^ and SARS‐COV‐2.^31^

In the current COVID‐19 pandemic, individuals with obesity have an increased risk of testing positive for SARS‐CoV‐2.^32^ Additionally, a substantial amount of patients hospitalized with COVID‐19 suffer from comorbidities closely associated with obesity, such as diabetes and cardiovascular disorders,^31^ presumably leading to the higher ICU admission rate and mortality rate of these patients with COVID‐19. Because most COVID‐19 cohort studies do not report whether patients with obesity also have metabolic disturbances related to MetS, although very likely, it is currently difficult to argue if and how MetS exacerbates the severity of COVID‐19 beyond the role of obesity. Proposed explanations for the strong association between obesity and severe COVID‐19 include pulmonary dysfunction,^33^ hypertension,^34^ upregulated angiotensin‐converting enzyme 2 (ACE2) expression,^35^, ^36^ hyperglycemia,^37^ dyslipidemia,^38^ insulin resistance,^39^, ^40^ chronic low‐grade inflammation,^33^ a pre‐existing pro‐thrombotic environment,^41^ and impairment of endothelial and gut barrier function.^42^, ^43^ Although in‐depth analysis of these mechanisms is beyond the scope of the current review, we refer to previously published mechanistic insights and other specific reviews when applicable. In this review, we aim to explain the molecular link between obesity and related metabolic disturbances and increased SARS‐CoV‐2 infection risk. We will focus specifically on upregulated TMPRSS2 and ACE2 expression (cell membrane proteins facilitating SARS‐CoV‐2 viral entry into host cells), hyperglycemia, and weakened immune surveillance. We then describe how several of the above‐mentioned mechanisms contribute to the poorer prognosis of COVID‐19 in this patient population. Lastly, the risk of reduced long‐term COVID‐19 vaccination efficacy in patients with obesity is discussed by focusing on potentially reduced memory T cell and memory B cell responses upon re‐infection.

## 2 OBESITY AND RELATED METABOLIC DISTURBANCES INCREASE THE RISK OF SARS‐COV‐2 INFECTION

Due to the variety of clinical symptoms associated with obesity, ranging from overexpression of proteins that facilitate viral entry into cells to hyperglycemia and hyperinsulinemia, these patients are at increased risk of acquiring infections, including SARS‐CoV‐2. In line, meta‐analysis data showed an association between individuals with obesity and the risk of testing positive for COVID‐19.^32^ Here, several factors that contribute to the increased susceptibility of patients with obesity and related metabolic disturbances to get infected with SARS‐CoV‐2 are described.

### 2.1 ACE2 and TMPRSS2 overexpression

**FIGURE 1:**
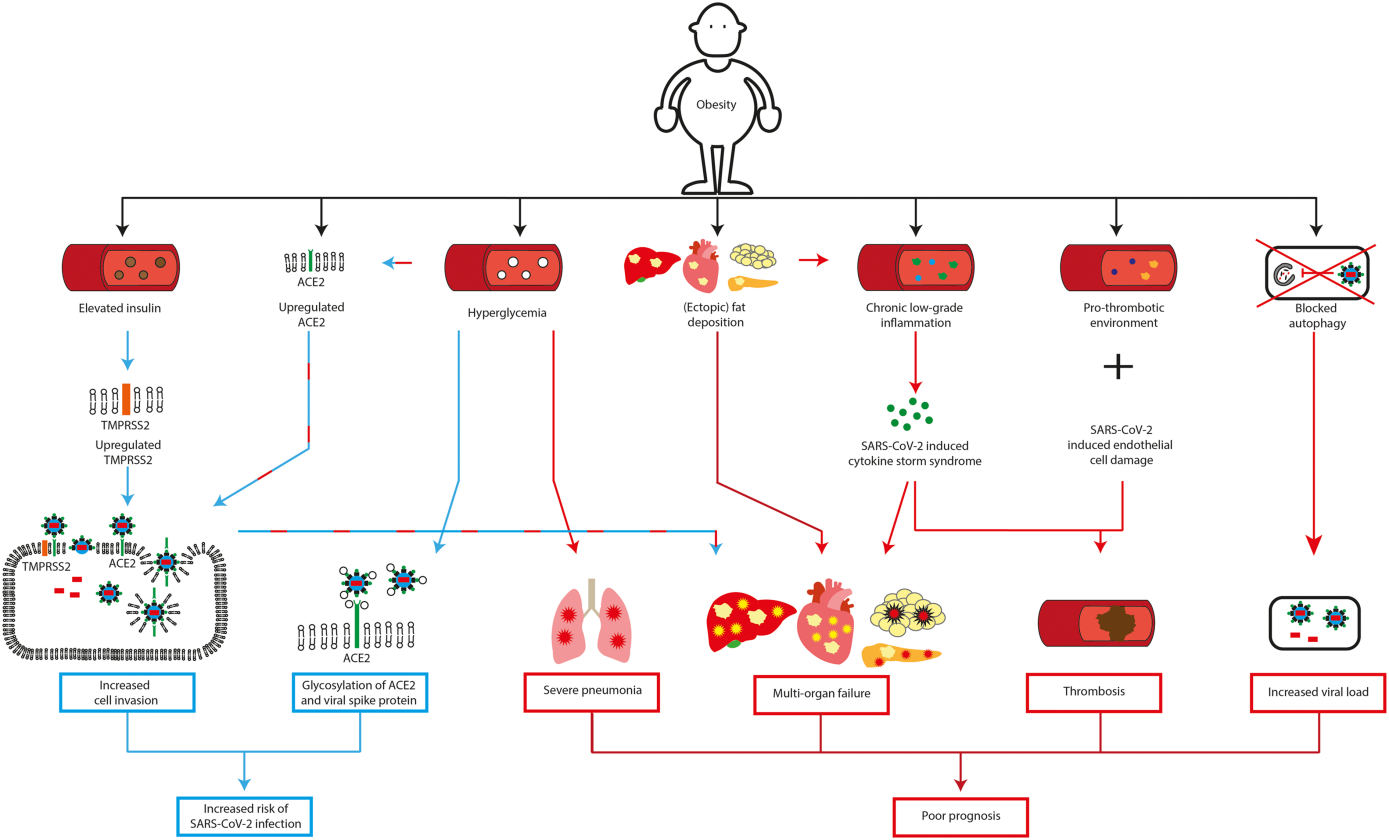
Obesity increases the risk of SARS‐CoV‐2 infection and leads to poorer prognosis. Blue arrows: Mechanisms contributing to increased SARS‐CoV‐2 infection risk in patients with obesity. Red arrows: Mechanisms contributing to poorer prognosis of COVID‐19 patients with obesity. In patients with obesity, elevated insulin levels upregulates TMPRSS2 expression, which in combination with overexpression of ACE2 increases SARS‐CoV‐2 cell invasion. Hyperglycemia increases ACE2 expression and potentially changes the glycosylation of ACE2 and the viral spike protein S, altering binding of S to ACE2. Together, these factors contribute to an increased risk of SARS‐CoV‐2 infection in patients with obesity. Hyperglycemia compromises the immune response in the lungs and may contribute to lung inflammation. Overexpression of ACE2 increases SARS‐CoV‐2 viral cell entry. Intracellularly, the virus induces cellular damage in adipose, heart, pancreas, and liver cells. In obesity, the organ function of the adipose tissue, heart, pancreas and liver is already affected due to (ectopic) fat deposition. SARS‐CoV‐2 can aggravate organ damage and thus contribute to multi‐organ failure. Chronic low‐grade inflammation, as observed in obesity, potentially facilitates the cytokine storm induced by SARS‐CoV‐2 consequently, extremely high levels of pro‐inflammatory cytokines, enhance cellular damage and eventually induce multi‐organ failure. Obesity is associated by a pro‐thrombotic environment. SARS‐CoV‐2 induces endothelial cell damage. In combination with the cytokine storm induced by SARS‐CoV‐2 this increases the risk of thrombosis in COVID‐19 patients with obesity. SARS‐CoV‐2 might block autophagy, resulting in decreased viral clearance. The disrupted autophagy process in obesity potentially further reduces SARS‐CoV‐2 clearance and by extension increases viral load. Together, these mechanisms lead to poorer prognosis of patients with obesity infected with SARS‐CoV‐2. ACE2, Angiotensin‐converting enzyme 2; TMPRSS2, transmembrane protease serine 2

After binding to host receptors, viruses can use the endosomal or the non‐endosomal pathway to enter host cells. Viral cell entry through the endocytic route is usually by transport in clathrin‐coated vesicles or pits, whereas the non‐endocytic route of entry involves directly crossing the plasma membrane at neutral pH.^44^ Generally, the endosomal pathway is advantageous for viruses: the endocytic vesicle supports intracellular transport of the virus, the endocytic vesicle contains specific proteases that provide necessary proteolytic activation of certain viruses, and upon endocytosis, no viral antigens remain on the cell membrane resulting in delayed detection by the immune system.^45^ Depending on the host cell type, SARS‐CoV‐2 can use the endosomal and non‐endosomal pathways to establish viral cell entry.^46^ In the non‐endosomal pathway of SARS‐CoV‐2, the spike protein (S) of virus binds to host receptor, angiotensin‐converting enzyme 2 (ACE2), a membrane bound aminopeptidase highly, but not exclusively, expressed in the lungs and cardiovascular system.^47^ Upon binding, the host protease, transmembrane protease serine 2 (TMPRSS2), expressed on the cell membrane, cleaves S into the S1 and S2 subunits to activate S to facilitate virus‐host cell fusion.^48^ In the endosomal pathway, SARS‐CoV‐2 also binds ACE2, but instead of activating TMPRSS2, SARS‐CoV‐2 is internalized via clathrin‐mediated endocytosis.^49^ Thus, in both pathways, ACE2 plays an essential role to induce viral cell invasion. In normal physiological situation, ACE2 functions as key regulatory enzyme in the renin‐angiotensin aldosterone system (RAAS), a hormonal system regulating blood pressure and water balance. In the RAAS system, angiotensin II (Ang II) binds to the angiotensin 1 or 2 receptor (AT1R or AT2R). Binding of Ang II to AT1R induces a proinflammatory vasoconstrictive effect, whereas binding of Ang II to AT2R counteracts this effect by promoting vasodilatation and inhibiting inflammatory events.^50^ ACE2 converts Ang II into its metabolite angiotensin‐(1‐7) (Ang (1‐7)). Following its conversion from Ang II, Ang (1‐7) then acts on the MAS1 proto‐oncogene G protein‐coupled receptor (MGRA) pathway, resulting in an anti‐inflammatory vasodilative response.^51^, ^52^ In the context of obesity and its related metabolic disturbances, elevated insulin levels, as observed in patient with insulin resistance and MetS, have been speculated to upregulate TMPRSS2 expression via phosphoinositide 3‐kinase/protein kinase B/androgen receptor signaling.^53^ In addition to TMPRSS2 overexpression, two ex vivo studies comparing heart samples from patients with (*n* = 23 and *n* = 40, respectively) and without (*n* = 9 and *n* = 15, respectively) heart failure found that ACE2 was upregulated in the heart failure samples.^54^, ^55^ Moreover, a large genome‐wide association study (74,124 type 2 diabetes cases and 824,006 controls) reported a causal link between type 2 diabetes and elevated ACE2 expression in the lung.^56^ A comprehensive description of altered ACE2 expression in comorbidities associated with severe COVID‐19 has recently been published.^57^ Strikingly, in patients with obesity and related metabolic disturbances, ACE2 expression can be upregulated not only due to the disease physiology but also due to the use of medication to control disease. For example, angiotensin receptor blockers, used to reduce blood pressure, can increase ACE2 expression^35^, ^36^, ^58^ and starting insulin therapy within 1 year of a diabetes diagnosis has been reported to be causally associated with increased ACE2 expression.^56^ Taken together, due to upregulated ACE2^54^, ^55^, ^56^, ^59^ and TMPRSS2^53^ expression in patients with obesity and related metabolic disturbances, SARS‐CoV‐2 cell invasion is facilitated, contributing to an increased risk to get infected with SARS‐CoV‐2 (Figure 1).

### 2.2 Hyperglycemia

Hyperglycemia, a hallmark of MetS and type 2 diabetes (both strongly associated with obesity), increases the rate of infections. Despite strict glycemic control, patients with diabetes still have a 1.7‐fold probability of developing an ICU‐acquired bloodstream infection compared with subjects without diabetes.^60^ Hyperglycemia potentially also contributes to an increased risk of SARS‐CoV‐2 infection.

Upon SARS‐CoV‐2 infection, lung epithelial cells start producing chemokines to recruit macrophages into the lung.^61^ When these macrophages also become virally infected with SARS‐CoV‐2, mitochondrial reactive oxygen species (mtROS) production is increased inducing stabilization of hypoxia‐inducible factor‐1α (HIF‐1α). Following, HIF‐1α promotes metabolic reprogramming of these macrophages driving them toward glycolysis. The metabolically reprogrammed glycolytic SARS‐CoV‐2 infected macrophages acquire a pro‐inflammatory phenotype resulting in an augmented secretion of interferons (IFN), leading to enhanced ACE2 expression in those lung macrophages.^62^ It is possible that due to elevated glucose levels, as frequently observed in obesity and MetS, the glycolysis‐mediated ACE2 upregulation in the lung macrophages of these patients subsequently increases SARS‐CoV‐2 cell invasion, contributing to an increased risk to get infected with SARS‐CoV‐2 (Figure 1).

In physiological conditions, glucose is not present in airway secretion. However, in a cohort study of patients admitted to a general ICU and expected to require intubation for more than 24 h (*n* = 60), a relationship appeared to be present between a high blood glucose concentration and the appearance of glucose in airway secretion. However, no correction was performed for confounding factors such as waist circumference or BMI.^63^ It has been suggested that glucose in airway surface liquid possibly contributes to airway inflammation by impairing host immunity through glycosylation of innate or acquired immune proteins.^63^ In the context of SARS‐CoV‐2 infection, it has also been proposed that hyperglycemia potentially changes the glycosylation of ACE2 and the viral spike protein S, altering binding of S to ACE2 and the degree of the immune response to the virus,^64^ thereby contributing to increased susceptibility of patients with MetS and type 2 diabetes (both strongly associated with obesity) to get infected with SARS‐CoV‐2 (Figure 1).

### 2.3 Weakened immune surveillance

Immunological surveillance, a monitoring process of the immune system to detect and destroy virally infected and neoplastically transformed cells in the body, is severely weakened in patients with obesity and related metabolic disturbances (see Andersen et al. for more mechanistic details).^28^ Obesity has been shown to be linked to a compromised B cell functioning, directly contributing to a weakened immune surveillance. For example, diet induced obesity (DIO) in mice lowers bone marrow B cell frequency, and in a murine infection study with influenza A viral infection, mice fed a western diet had suppressed antibody titers compared with mice receiving a control diet.^65^ In line, a (small) human cohort study comparing four groups (young lean *n* = 8; young obese *n* = 6; elderly lean *n* = 8; and elderly obese *n* = 4) indicated that obesity is associated with reduced antibody titers after influenza vaccination, in both young and elderly patients.^66^ In addition to a reduced humoral immune response, available evidence also indicates that impaired cellular immunity contributes to weakened immune surveillance in obesity. In that context, progressive obesity in mice is linked to inhibition of thymopoiesis, consequently restricting T cell repertoire diversity. This observation correlates with data in middle‐aged humans, which also indicated that obesity compromises thymic output.^23^ Moreover, a human cohort study (comparing individuals with healthy weight *n* = 137, overweight *n* = 154, and obesity *n* = 164) demonstrated that individuals with obesity have decreased effector memory T (T_em_) cell activation after ex vivo vaccine strain virus challenge compared with healthy weight individuals.^67^ Remarkably, initial clinical trials show that COVID‐19 vaccines are effectively protecting patients with obesity and patients with obesity‐related metabolic disturbances (e.g., chronic pulmonary diseases, diabetes, hypertension, liver disease, and cardiovascular disease) in the short‐term.^68^, ^69^, ^70^, ^71^ These most recent data indicate that SARS‐CoV‐2 is well detected by immune surveillance mechanisms in patients with obesity and related metabolic disturbances. As such, these findings are suggesting that a potentially reduced long‐term COVID‐19 vaccination efficacy in obesity (cfr. infra) would not be due to defects in immune surveillance mechanisms in these vulnerable patients.

## 3 COVID‐19 LEADS TO POORER PROGNOSIS IN PATIENTS WITH OBESITY AND RELATED METABOLIC DISTURBANCES

In the current COVID‐19 pandemic, patients suffering from diabetes, hypertension, and cardiovascular disorders are overly represented in the hospital.^31^, ^72^ This observation is indicative of these patients being a vulnerable population with increased risk of complications and poorer prognosis upon SARS‐CoV‐2 infection.

### 3.1 Pneumonia

The most frequent serious complication of COVID‐19 is pneumonia. Upon hospital admission (almost) all patients suffer from unilateral or bilateral pneumonia.^9^, ^73^ Upon SARS‐CoV‐2 infection, lung macrophages undergo a HIF‐1α mediated metabolic reprogramming toward glycolysis, inducing a pro‐inflammatory phenotype.^62^ These glycolytic macrophages secrete pro‐inflammatory cytokines, such as TNFα and IL‐6, which have been suggested to contribute to epithelial cell death in the lungs of patients with COVID‐19.^62^ It is possible that due to elevated glucose levels, as observed in obesity, MetS, and type 2 diabetes, the glycolytic macrophages secrete enhanced levels of pro‐inflammatory cytokines. As such, high blood glucose levels, may increase lung inflammation, predisposing patients with obesity‐related metabolic disturbances to become more susceptible to develop severe pneumonia upon SARS‐CoV‐2 infection, which potentially partially explains the poorer prognosis of these patients upon SARS‐CoV‐2 infection (Figure 1).

### 3.2 Multi‐organ failure

Apart from the lungs, SARS‐CoV‐2 also affects other organs. COVID‐19 has been shown to be associated with gastrointestinal injury, reduced kidney function, and liver injury (see Renu et al. for more details).^74^ Here, we focus specifically on adipose tissue, heart, liver, and pancreas dysfunction in COVID‐19 patients with obesity and related metabolic disturbances.

#### 3.2.1 Multi‐organ failure (adipose tissue)

Central or visceral obesity is defined as increased adipose tissue surrounding the intra‐abdominal organs. Ironically, despite the increased health risk associated with visceral obesity (e.g., development of cardiovascular diseases, insulin resistance and diabetes), patients with obesity may have prognostic benefits in some diseases such as heart failure,^75^ chronic obstructive pulmonary disease (COPD),^76^ and pneumonia,^77^ being expressed as the “obesity paradox.” However, recent meta‐analysis data reveal no evidence for an “obesity paradox” in the context of COVID‐19.^78^ As extensively reviewed by Kruglikov et al.^79^ and Goossens et al.,^80^ one of the main reasons for poor prognosis of COVID‐19 patients with visceral obesity is an increased number of adipocytes in combination with upregulated ACE2 expression. This turns the visceral adipose tissue into a viral reservoir for SARS‐CoV‐2, eventually leading to visceral adipose tissue dysfunction and systemic inflammation,^79^, ^81^ presumably contributing to the poorer prognosis COVID‐19 patients with obesity (Figure 1).

#### 3.2.2 Multi‐organ failure (heart)

ACE2, the receptor through which SARS‐CoV‐2 enters host cells, is upregulated in the heart of patients with cardiovascular disorders^54^, ^55^ (frequently observed in patients with obesity). This makes the heart a potential target organ for SARS‐CoV‐2 infection. Tissue distribution analysis of ACE2 in human donor hearts through single‐cell RNA sequencing revealed that ACE2 is highly expressed in pericytes, whereas cardiomyocytes only have a low expression. Furthermore, cell–cell interaction analysis between pericytes and other cell types indicated that neuron‐like cells and endothelium cells (ECs) have the closest crosstalk interaction with pericytes. Therefore, it is suggested that SARS‐CoV‐2 attacks pericytes and causes capillary ECs dysfunction, leading to micro‐circulation disorders.^55^ Moreover, cardiac pericyte dysfunction may increase the propensity for atrial fibrillation via increased myocardial inflammation, fibrosis, increased tissue edema, and interstitial hydrostatic pressure.^82^

Because in visceral adipocytes of patients with central obesity ACE2 is upregulated^79^, ^81^ and meta‐analysis data showed a strong positive correlation between epicardial adipose tissue thickness and MetS,^83^ ACE2 expression in epicardial fat cells from patients with MetS has also been explored. Data are limited, but one study analyzing epicardial biopsies from patients who underwent open‐heart surgery demonstrated that, similar to visceral adipocytes, epicardial fat cells highly express ACE2. Relevantly, highest ACE2 expression was found in biopsies from patient suffering from obesity and diabetes.^84^ Therefore, the thickened epicardial adipose tissue, observed in patients with obesity‐related insulin resistance,^85^ diabetes,^84^ and MetS^83^ might be considered another viral reservoir for SARS‐CoV‐2. Moreover, SARS‐CoV‐2 internalizes ACE2 upon binding, resulting in loss of cell surface ACE2.^43^ Because loss of ACE2 expression in epicardial adipose tissue has been associated with increased epicardial adipose tissue inflammation,^86^ a mechanistic link with COVID‐19 related myocarditis^87^ might be present. Furthermore, it is tempting to speculate that fat deposition, presence of perivascular adipocytes in the heart, and adipocyte infiltration into the myocardium contribute to COVID‐19‐related heart damage in patient with obesity or related metabolic disturbances by inducing pro‐inflammatory responses; however, there are currently no solid data available to support this.

Overall, because patients with obesity or related metabolic disturbances often already have a reduced heart function, accumulative heart damage induced by cardiac pericyte dysfunction and increased myocardial inflammation may well contribute to poorer prognosis of these patients upon SARS‐CoV‐2 infection (Figure 1).

#### 3.2.3 Multi‐organ failure (liver)

Results from a recent (relatively small) cohort study comparing patients with obesity (*n* = 20) and patients with obesity and NASH (*n* = 17) demonstrated that ACE2 and TMPRSS2 are upregulated in the liver of patients with NASH,^88^ making the liver another potential target organ for SARS‐CoV‐2 infection. In line with these observations, single‐cell RNA sequencing of healthy liver tissue to investigate the tissue distribution of hepatic ACE2 demonstrated high ACE2 expression specifically in the cholangiocytes, whereas low or no ACE2 expression is observed in the hepatocytes, immune cells, and stromal cells. Therefore, it can be speculated that SARS‐CoV‐2 uses ACE2 as host receptor to induce direct damage of the bile ducts, leading to liver damage and reduced liver function.^89^ For example, a cohort study with patients hospitalized with COVID‐19 (*n* = 1099) demonstrated that around 20% of the patients has elevated serum aspartate aminotransferase (AST) and alanine aminotransferase (ALT), and around 10% has elevated serum total bilirubin upon hospital admission.^90^ Similarly, another cohort study (*n* = 148) reported that 37.2% of the patients hospitalized with COVID‐19 have an abnormal liver function, indicated by elevated ALT, AST, g‐glutamyltransferase, alkaline phosphatase (ALP), and total bilirubin.^91^ Elevated serum ALP levels are indicative for bile duct damage, supporting the suggestion that SARS‐CoV‐2 uses ACE2 on the cholangiocytes to induce direct damage of the bile ducts, leading to reduced liver function. Yet, in both these cohort studies, it is not clear whether the COVID‐19 patients with liver damage upon hospital admission were obese or had obesity‐related metabolic disturbances. Also, in two most recent meta‐analysis, which confirm that COVID‐19 affects liver function, a split group analysis of patients with and without obesity is missing.^92^, ^93^ Moreover, these meta‐analysis do not distinguish between direct damage of the hepatocytes consequently reducing liver function or SARS‐CoV‐2 induced cholangiocytes damage eventually affecting liver function.

Given that COVID‐19 negatively affects liver function, and obesity‐associated NASH is also characterized by reduced liver function, the latter group potentially forms a high‐risk group with poor prognosis upon SARS Cov‐2 infection. In line, preliminary data from a multicenter cohort study (*n* = 153) reported that pre‐existing chronic liver disease (22.4% non‐alcoholic fatty liver disease [NAFLD], 19.7% alcohol, 11.8% hepatitis B, 10.5% hepatitis C, 35.6% other/combination) appears to be an independent risk factor for poor outcome in COVID‐19 patients, also after correction for BMI in multiple logistic regression analysis.^94^ One of the universal mechanisms affecting liver function in patients with obesity is excessive ectopic fat accumulation in the liver. Excessive lipid deposition in the liver exacerbates hepatic insulin resistance and promotes inflammation. Relevantly, an ongoing prospective COVID‐19 cohort study (currently *n* = 201, 18% hospitalized) reported that ectopic fat in the liver was higher in individuals hospitalized with COVID‐19 compared with non‐hospitalized individuals,^95^ indicating a potential link between hepatic steatosis and severe COVID‐19. Presumably, the low‐grade chronic inflammatory state in patients with obesity‐associated NAFLD or NASH aggravates the immunogenic response induced upon SARS‐CoV‐2 infection. Overall, elevated ACE2 levels in the liver of patients with obesity and related metabolic disturbances, together with exacerbated pro‐inflammatory responses as a consequence of hepatic steatosis, may well explain the poorer prognosis of these patients upon SARS‐CoV‐2 infection (Figure 1).

#### 3.2.4 Multi‐organ failure (pancreas)

ACE2 is expressed in the pancreas, particularly in the exocrine glands and the islets of the pancreas, as demonstrated by a tissue distribution study utilizing single‐cell RNA sequencing.^96^ In line, SARS‐CoV‐2 exposure to ex vivo cultured human pancreatic islets isolated from human donors, resulted in viral SARS‐CoV‐2 replication, and affected glucose‐dependent insulin secretion in the pancreatic islets.^97^ Although clinical data are limited, case reports of new‐onset type 1 diabetes after SARS‐CoV‐2 infection have been described,^98^, ^99^ and results from an early cohort study with severely ill COVID‐19 patients (*n* = 121) demonstrated that 17% (13 out of 121) of those patients has pancreatic injury, although four of these patients had been treated with glucocorticoids during hospitalization, which may be associated with drug‐induced pancreatitis.^96^ In addition, two COVID‐19 cohort studies (*n* = 218 and *n* = 50, respectively) indicated that a selection of patients with type 2 diabetes, who presented with diabetic ketoacidosis upon hospital admission, showed high mortality rates.^100^, ^101^ Together, these data suggest that SARS‐CoV‐2 indeed potentially induces pancreatic damage, which might have detrimental consequences in patients with pre‐existing pancreatic diseases.

Obesity is characterized by excessive lipid deposition in the pancreas, which is associated with impaired insulin secretion^102^ and inflammation.^103^ Crucially, the same prospective cohort study (currently *n* = 201, 18% hospitalized) comparing ectopic fat in the liver between hospitalized and non‐hospitalized COVID‐19 patients also reported that ectopic fat in the pancreas was higher in individuals hospitalized with COVID‐19 compared with non‐hospitalized individuals,^95^ indicating a potential link between excessive ectopic fat deposition in the pancreas and severe COVID‐19. The chronic low‐grade inflammatory state in patients with obesity or related metabolic disturbances may exacerbate the immunogenic response induced upon SARS‐CoV‐2, consequently aggravating pre‐existing pancreatic inflammation, thereby also contributing to the poorer prognosis of patients with obesity upon SARS‐CoV‐2 infection (Figure 1).

### 3.3 Cytokine storm syndrome

Chronic low‐grade inflammation is a well‐established characteristic in patients with “metabolically unhealthy” obesity. Mechanisms contributing to this chronic low‐grade inflammatory state include increased activation of the RAAS system,^80^ in which specifically Ang II has been shown to induce metabolic inflammation^104^ and altered adipose tissue functioning results in an amplified release of pro‐inflammatory adipocytokines.^105^ In that context, elevated leptin levels (which might reduce long‐term COVID‐19 vaccination efficacy [cfr. infra]), the driving force for obesity‐related metabolic disorders,^106^ can contribute to chronic low‐grade inflammation.^107^ Moreover, lipid metabolism is severely dysregulated in obesity. This dysregulated lipid metabolism, in combination with elevated oxidative stress levels (which potentially also affects long‐term COVID‐19 vaccination efficacy in patients with obesity [cfr. Infra]), leads to an increased production of oxidized low‐density lipoproteins (oxLDL). Subsequently, increased oxLDL contributes to chronic low‐grade inflammation by interacting with immune cells and disturbing cholesterol trafficking.^108^ As such, increased oxLDL levels strongly correlate with obesity‐related metabolic disturbances.^108^, ^109^ Similarly to obesity, COVID‐19 is also associated with an excess production of pro‐inflammatory cytokines.^110^ SARS‐CoV‐2 infection of epithelial cells mediates mitochondrial ROS production, consequently stimulating NLR family pyrin domain containing 3 (NLRP3) and nuclear factor kappa‐light‐chain‐enhancer of activated B cells (NF‐kB) synthesis, which triggers excessive cytokine release by immune cells.^111^ Moreover, SARS‐CoV‐2 enhances this cytokine secretion by internalizing ACE2 upon binding, due to which Ang II cannot be converted anymore, leading to more cytokine production.^43^, ^80^ Crucially, there seems to be a direct link between obesity‐mediated chronic low‐grade inflammation and the cytokine storm development upon SARS‐CoV‐2 infection observed in these patients: upon SARS‐CoV‐2 infection, macrophages chronically exposed to oxLDL are suggested to potentiate pre‐existing chronic low‐grade inflammation through apoptosis‐associated speck‐like protein containing a CARD (ASC) mediated caspase‐1 activation, leading to hyper‐inflammation and excessive cytokine secretion.^112^ In addition, SARS‐CoV‐2 can interact with protein kinase R (PKR) and PKR‐like endoplasmic reticulum kinase (PERK),^113^ potentially downregulating the insulin signaling pathway through serine phosphorylation of insulin receptor substrates, eventually enhancing insulin resistance.^39^ Insulin resistance in adipocytes results in production of monocyte chemoattractant protein 1 (MCP1), which recruits pro‐inflammatory macrophages, creating a pro‐inflammatory environment.^114^ Because insulin resistance is associated with obesity, and creates a pro‐inflammatory environment, it might be possible that the cytokine storm observed in COVID‐19 patients with obesity is aggravated via SARS‐CoV‐2‐induced insulin resistance.^39^ Overall, chronic low‐grade inflammation and insulin resistance potentially facilitate the cytokine storm induced by SARS‐CoV‐2. Consequently, extremely high levels of pro‐inflammatory cytokines enhance cellular damage and eventually induce multi‐organ failure, thereby contributing to poorer prognosis of this COVID‐19 infected patient population (Figure 1).

### 3.4 Thromboembolism

Upon entry of SARS‐CoV‐2 into endothelial cells, inflammatory responses are induced thereby generating a pro‐thrombotic environment.^115^ Extensive meta‐analysis has demonstrated that COVID‐19 patients with thromboembolism have a higher ICU admission rate and mortality rate compared with patients without thromboembolism.^116^ In the context of obesity and related metabolic disturbances, several correlates exist between increased fibrinogen, factor VIII, von Willebrand factor, plasminogen activator inhibitor‐1 (PAI‐1), and decreased antithrombin III with metabolic features such as waist circumference, BMI, liver tests, and parameters of lipid and glucose metabolism as reported in a cohort of patients with NAFLD (*n* = 273). After multiple regression analysis, hepatic steatosis remained an independent predictor of PAI‐1 levels in this cohort.^117^ Also, other cohort studies (*n* = 49 and *n* = 60) are clearly pointing toward an increased risk of thrombosis in COVID‐19 patients with obesity. For instance, anti‐thrombin levels were reported to be significantly lower in COVID‐19 patients with central obesity compared with patients without obesity,^118^ whereas D‐dimer levels were significantly higher. Elevated D‐dimer was independently associated with ALT elevation,^119^ indicating that microvascular thrombosis might be induced by liver inflammation in these patients. In line, another cohort of COVID‐19 patients with (*n* = 75) and without (*n* = 125) NALFD reported that NAFLD was associated with elevated D‐dimer levels at the time of ICU admission, and crucially, within this cohort, the incidence of deep vein thrombosis was higher in COVID‐19 patients with NAFLD.^120^ Previously described pro‐inflammatory mechanisms (e.g., excessive ectopic fat deposition in the liver and the cytokine storm syndrome) contribute to extremely high levels of pro‐inflammatory cytokines, which consequently, can activate the coagulation system.^115^, ^121^ Presumably, this response enhances the generation of the pro‐thrombotic environment induced upon SARS‐CoV‐2 entry into endothelial cells, subsequently increasing the risk of thrombosis in COVID‐19 patients with MetS and potentially contributing to the higher ICU treatment rate and mortality rate of these patients upon SARS‐CoV‐2 infection (Figure 1).

### 3.5 Viral load

In normal physiological conditions, autophagy contributes to the defense against viral infections. Double membrane vesicles containing viral particles fuse with lysosomes where the virus is degraded. The innoxious viral antigens are used for antigen presentation to T cells to coordinate a powerful adaptive immune response against the virus. Additionally, autophagy initiates an innate immune response by activating pattern recognition receptor signaling resulting in IFN production.^122^ However, in patients with obesity, MetS, cardiovascular disease, or NASH, the autophagy process shows apparent abnormalities.^123^, ^124^ For instance, in MetS, autophagy is attenuated in the liver, whereas in adipose tissue, the autophagic activity is induced.^124^ One of the factors contributing to attenuation of the autophagy activity in NASH is an elevation of plasma cholesterol. Cholesterol accumulation inside lysosomes leads to lysosomal dysfunction and inhibition of autophagy. A possible explanation for the blocked autophagy is that lipid accumulation alters the membrane structure, consequently leading to improper fusion between lysosomes and autophagosomes.^125^, ^126^ In the context of host defense against SARS‐CoV‐2, this blocked autophagy process potentially contributes to decreased SARS‐CoV‐2 clearance, consequently leading to increased viral load. Additionally, many viruses have acquired properties to manipulate autophagy for their own benefit.^122^, ^127^ In line, SARS‐CoV‐2 has been shown to reprogram host cell metabolism to limit AMP‐activated protein kinase/mammalian target of rapamycin complex 1 (AMPK/mTORC1) activation and autophagy.^128^ Mechanistical details on how disturbed autophagy potentially contribute to the close correlation between obesity, and the severe clinical manifestations of COVID‐19 have recently been reviewed elsewhere.^129^ Overall, in patients with MetS and NASH the disrupted autophagy process potentially reduces SARS‐CoV‐2 clearance further and by extension increases viral load, leading to more severe COVID‐19 symptoms and poor prognosis upon SARS‐CoV‐2 infection (Figure 1).

## 4 OBESITY POTENTIALLY REDUCES LONG‐TERM COVID‐19 VACCINATION EFFICACY

Although clinical care for patients with COVID‐19 has significantly improved last few months, there are currently no therapies proven to be effective to cure COVID‐19. Thus, there remains an urgent need for vaccines to protect vulnerable populations, including patients with obesity and associated metabolic comorbidities. The European Medicine Agency (EMA) and The US Food and Drug Administration (FDA) have both authorized two mRNA‐based COVID‐19 vaccines for all adult individuals.^68^, ^69^, ^130^ In addition to these two mRNA‐based COVID‐19 vaccines, the FDA^130^ and EMA^70^ have recently authorized other adenovirus based COVID‐19 vaccines, although these vaccines are currently on hold in several countries due to thrombosis related complications in some patients. Moreover, other potential COVID‐19 vaccine candidates are currently in development.^131^ Obesity is associated with a reduced immunogenicity in response to vaccination for hepatitis B, tetanus, and influenza.^23^, ^24^, ^28^, ^29^ These vaccination studies hardly report whether patients with obesity also have MetS‐related metabolic disturbance. Therefore, although very likely, we cannot argue how MetS reduces immunogenicity after vaccination beyond the role of obesity. Here, we discuss how patients with obesity potentially develop a reduced immunogenicity in response to COVID‐19 vaccination by focusing on memory T cell and memory B cell responses in these patients.

### 4.1 Memory T cells

**FIGURE 2:**
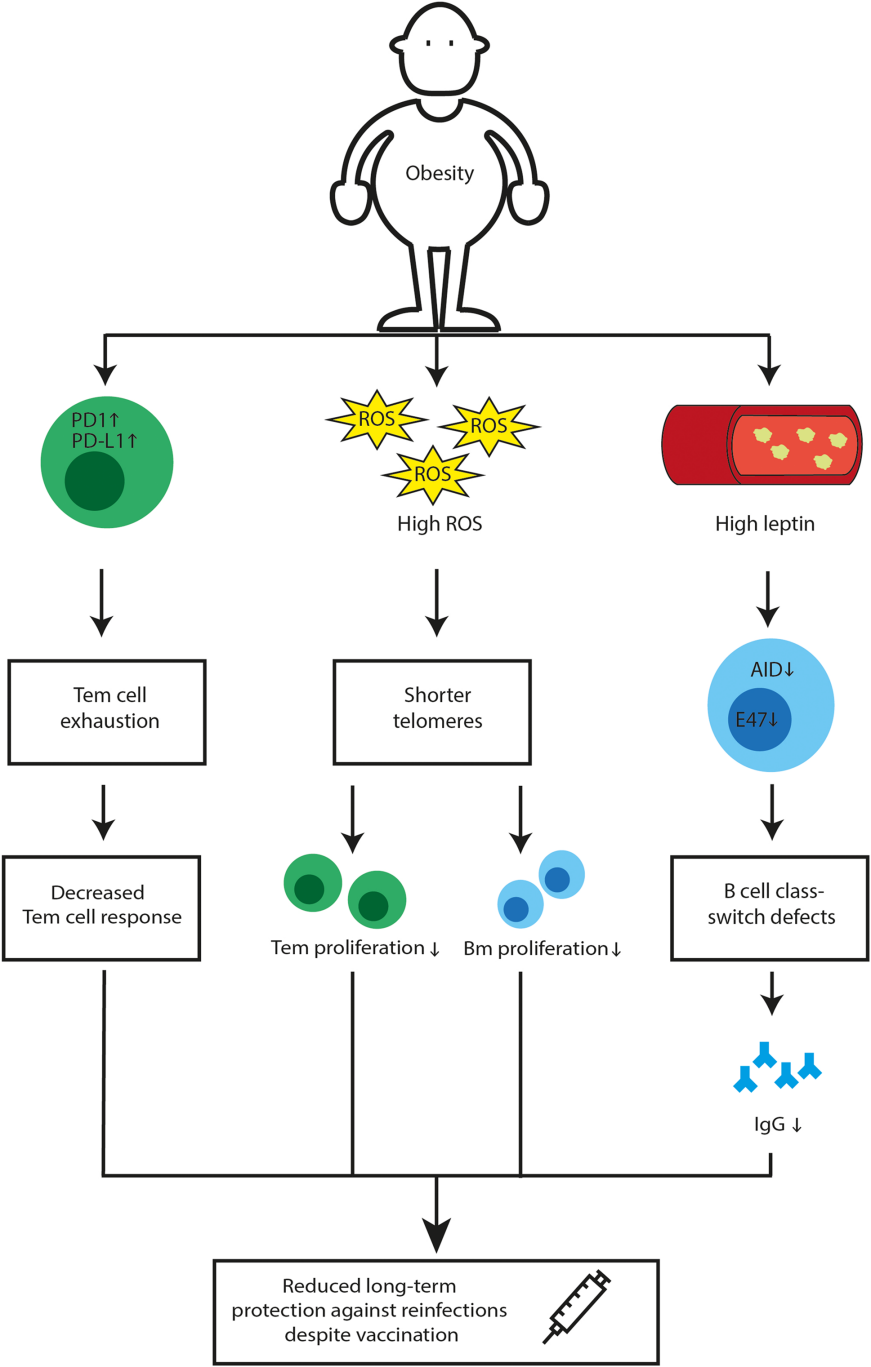
Obesity potentially reduces long‐term COVID‐19 vaccination efficacy. Obesity alters PD1 and PD‐L1 expression on T_em_ cells, weakening stem cell responsiveness. Also, obesity is associated with elevated systemic ROS causing shorter telomere length of immune cells, leading to decreased T_em_ and B_m_ proliferation. Moreover, high serum leptin levels, as observed in obesity, reduces AID and E47 expression in B cells, inducing B cell class‐switching defects, potentially leading to decreased SARS‐CoV‐2‐specific‐IgG production. These factors together reduce long‐term protection against re‐infections. Therefore, despite COVID‐19 vaccination, patients with obesity may still be vulnerable for re‐infection with SARS‐CoV‐2. AID, activation‐in induced cytidine deaminase; Bm, B memory cells; IgG, immunoglobulin G; PD1, programmed cell death protein 1; PD‐L1, programmed death‐ligand 1; ROS, reactive oxygen species; T_em_ effector memory T cells

The cytotoxic T cell response is essential for the viral infection clearance, and effector memory T (T_em_) cells play a crucial role in providing long‐term immunity.^132^ A recent cohort study (*n* = 36) indicated that after 17 years, blood collected from individuals recovered from SARS still contains long‐lived T_em_ cells reactive against SARS‐CoV peptides. Remarkably, these long‐lived T_em_ cells also cross‐react to proteins of SARS‐CoV‐2.^133^ Other cohort studies have found SARS‐CoV‐2 specific T cells,^134^, ^135^ and recently, SARS‐CoV‐2‐specific T_em_ cells^136^ could be isolated from blood of patients recovered from COVID‐19 (*n* = 20, *n* = 25, and *n* = 235, respectively). These SARS‐CoV‐2 specific T_em_ cells, which are generated upon primary infection, or upon injection with a COVID‐19 vaccine,^137^, ^138^ are needed for long‐term protection against SARS‐CoV‐2. Unfortunately, several animal studies indicate that T_em_ cell responsiveness is severely hampered in obesity. For instance, after primary influenza infection, both lean and DIO mice have a population of influenza specific T_em_ cells in the lung, but obese mice show a greater percent loss of T_em_ cells over time, resulting in significantly decreased T_em_ cell numbers in the lung of obese mice post infection.^139^ It has also been shown that after a secondary influenza virus challenge, although DIO mice had similar absolute percentages of T_em_ cells in the lung compared with lean mice, the percentage of influenza specific T_em_ cells responding to the challenge by producing IFNy was significantly reduced.^140^ Also, after secondary influenza infection, T_em_ cells from DIO mice have an altered cellular metabolism, largely characterized by increased oxygen consumption, which was not reversed with weight loss.^141^ However, because other mice studies did not find an effect of obesity on T_em_ cell development and function after influenza infection or vaccination,^142^, ^143^ controversy exists about the impact of obesity on T_em_ cell responsiveness after infection or vaccination. Yet a human cohort study comparing individuals with normal weight (*n* = 137) and obesity (*n* = 164) vaccinated against influenza demonstrated that individuals with obesity have decreased T_em_ cell activation after ex vivo vaccine strain virus challenge.^67^ Overall, these data seem to suggest that patients with obesity potentially have a reduced T_em_ cell responsiveness, even after weight loss. The underlying mechanisms of the observed reduced T_em_ cell responsiveness after infection or vaccination is still unknown, but shorter telomere length of T cells might offer a possible explanation.^144^ In a cohort of 22 elderly (>70 years) vaccinated against influenza, patients with long T cell telomeres had a slight increase in the percentage of influenza‐specific T cells in their blood compared with elderly with a shorter T cell telomere length, 84 days post vaccination.^145^ Because obesity is associated with shorter telomeres due to chronic low‐grade inflammation and oxidative stress,^146^, ^147^ it is tempting to speculate that this affects T_em_ cell proliferation and development upon infection and vaccination. Another explanation might be, similar as in cancer, that obesity increases the expression of programmed cell death protein 1 (PD‐1) and programmed death‐ligand 1 (PD‐L1) on T_em_ cells inducing T cell exhaustion and thus reducing T_em_ cell responsiveness,^148^ which by extension potentially diminishes long‐term protection against re‐infections. In the context of COVID‐19, initial clinical trials show that both mRNA‐based COVID‐19 vaccines and the adenovirus based COVID‐19 vaccines are effectively protecting humans in short‐term, also when suffering from obesity or associated metabolic disturbances.^68^, ^69^, ^71^ Nonetheless, for all vaccines, it is currently not known yet how long this protection will last. Because obesity is associated with reduced T_em_ cell responsiveness, long‐term protection against re‐infections is also diminished. Therefore, despite COVID‐19 vaccination, patients with obesity may still be more vulnerable for re‐infection with SARS‐CoV‐2 (Figure 2).

### 4.2 Memory B cells

The humoral B cell immune response is crucial for clearance of viral infections and long‐term immunity.^149^ The SARS‐CoV‐2 virus elicits a robust humoral B cell response as evidenced by a high production of virus specific antibodies found in blood of several COVID‐19 cohorts (*n* = 518 and *n* = 607).^150^, ^151^ A recent study showed that most individuals reach a neutralizing antibody peak after an average of 23.1 days post onset of symptoms. In the follow‐up period of this study, it was found that IgM and IgA antibodies decline rapidly after 20–30 days post onset of symptoms, whereas IgG antibodies lasted longer (max study follow‐up of 95 days).^152^ Relevantly, a longitudinal cohort study (*n* = 254) showed that during the early phase, SARS‐CoV‐2 specific class‐switched IgG and IgM memory B (B_m_) cells are similarly present in high amounts in the blood of patients recovered from COVID‐19, whereas the population of class‐switched IgA B_m_ cells was low. Over time, the class‐switched IgM B_m_ cell population declines and the class‐switched IgG B_m_ cells become the dominant population.^153^ Notably, these SARS‐CoV‐2 specific class‐switched B_m_ cells, either induced by primary infection or upon vaccination,^154^, ^155^ are involved in regulating the immune response against SARS‐CoV‐2 upon re‐infection. However, similar as T_em_ cell responsiveness, several studies indicate that humoral B_m_ cell responsiveness is also reduced in obesity. For example, compared with lean mice, DIO mice revealed lower hemagglutination inhibition (HAI) titers (standard assay used to determine antibody levels to influenza virus) after influenza infection^65^, ^143^ or adjuvant influenza vaccination.^156^ A similar result was found in a human cohort study (consisting of four groups, young lean *n* = 8; young obese *n* = 6; elderly lean *n* = 8 and elderly obese *n* = 4) comparing individuals with normal weight and obesity vaccinated against influenza, in which both the young and elderly individuals with obesity had a decreased percentage of class‐switched B_m_ cells and an increased percentage of exhausted B_m_ cells in their blood, compared with respective healthy weight individuals of the same age.^66^ These observations potentially partially explain, the increased risk of acquiring influenza infection, despite vaccination, of individuals with obesity compared with individuals with a healthy weight. The data may be explained by increased leptin levels (a pro‐inflammatory adipokine) observed in individuals with obesity.^157^ In the presence of leptin, ex vivo cultured human B cells showed class‐switching defects, potentially regulated via leptin‐induced downregulation of activation‐induced cytidine deaminase (AID) (the enzyme necessary for class switch recombination, somatic hypermutation and IgG production) and its transcriptional regulator E47. Therefore, leptin might decrease influenza‐vaccine‐specific‐IgG production in individuals with obesity.^158^ In contrast, other cohort studies comparing individuals with a normal weight and obesity vaccinated against seasonal influenza (*n* = 34, 50% obese)^67^ or tick‐borne encephalitis (*n* = 73, 50% obese),^159^ indicated that individuals with obesity at first develop stronger antibody responses but have a steeper decline over time. In addition, individuals with obesity vaccinated against tick‐borne encephalitis had more naïve B cells in their blood and less expansion to B_m_ cells upon booster vaccination.^159^ Similarly, as discussed above, this decreased B_m_ cell expansion might also be explained by the shorter telomere length in obesity.^145^, ^146^, ^147^ In the context of COVID‐19, initial clinical trials show that al authorized COVID‐19 vaccines are effectively protecting patients with obesity and related metabolic disturbances in the short‐term.^68^, ^69^, ^70^, ^71^ Nonetheless, for these vaccines, it is currently not known yet how long this protection lasts in healthy individuals as well as in patients with obesity. Because obesity is associated with reduced humoral B_m_ cell responsiveness, long‐term protection against re‐infections is also diminished. Therefore, despite COVID‐19 vaccination, patients with obesity may still be vulnerable for re‐infection with SARS‐CoV‐2 (Figure 2).

## 5 CONCLUSION AND FUTURE DIRECTIONS

Overall, due to increased expression of proteins facilitating viral entry into cells and hyper‐glycosylation of those proteins, patients with obesity and related metabolic disturbances have an increased risk of becoming infected with SARS‐CoV‐2. In addition, due to a compromised immune response in the lungs, hyper‐inflammatory systemic responses, increased risk of thrombosis and increased viral load, patients with obesity and related metabolic disturbances also develop severe complications upon SARS‐CoV‐2 infection, leading to higher morbidity and mortality risks upon COVID‐19. Although clinical care for patients with COVID‐19 has significantly improved last few months, there are currently no therapies proven to be effective to cure COVID‐19. Therefore, there remains an urgent need for vaccines to protect vulnerable populations such as patients with obesity and related metabolic disturbances. Initial clinical trials show that currently authorized COVID‐19 vaccines are effectively protecting these patients, nonetheless, it is currently not known yet how long this protection lasts.^68^, ^69^ Obesity is associated with reduced memory immune responses leading to diminished long‐term protection against re‐infections. Therefore, despite encouraging COVID‐19 vaccination results, patients with obesity may still be vulnerable for re‐infection with SARS‐CoV‐2 in the long run. This may affect herd immunity and impact SARS‐CoV‐2 elimination. In conclusion, to limit further impact of COVID‐19 on patients with obesity and related metabolic disturbances, and society, long‐term COVID‐19 vaccine efficacy should be closely monitored in these patients.

## CONFLICT OF INTERESTS

All authors certify that they have no affiliations with or involvement in any organization or entity with any financial interest (such as honoraria; educational grants; participation in speakers' bureaus; membership, employment, consultancies, stock ownership, or other equity interest; and expert testimony or patent‐licensing arrangements), or non‐financial interest (such as personal or professional relationships, affiliations, knowledge or beliefs) in the subject matter or materials discussed in this manuscript.
